# Robotic Resection With Hyperthermic Intrathoracic Perfusion Chemotherapy for Thymoma Recurrence

**DOI:** 10.1155/crom/1802396

**Published:** 2026-09-12

**Authors:** Paula Duarte D′Ambrosio, Gustavo Schvartsman, Mathias de Souza Ribeiro, Enzo Campedelli Borges, Marina Cardoso de Castro, Paulo Manuel Pêgo Fernandes, Ricardo Mingarini Terra

**Affiliations:** ^1^ Departamento de Cirurgia Toracica, Hospital Sirio Libanes, Brasília, Distrito Federal, Brazil, hospitalsiriolibanes.org.br; ^2^ Departamento de Cirurgia Toracica, Hospital Israelita Albert Einstein, São Paulo, São Paulo, Brazil, einstein.br; ^3^ Universidade de Santo Amaro, São Paulo, Brazil, unisa.br; ^4^ Faculdade Israelita de Ciências da Saúde Albert Einstein, São Paulo, Brazil; ^5^ Instituto do Coração, Hospital das Clínicas HCFMUSP, Faculdade de Medicina, Universidade de São Paulo, São Paulo, São Paulo, Brazil, usp.br

## Abstract

Thymoma recurrence with pleural dissemination remains a therapeutic challenge, often requiring multimodal treatment strategies. Cytoreductive surgery combined with hyperthermic intrathoracic chemotherapy (HITHOC) has emerged as a promising approach for selected patients; however, reports using minimally invasive robotic platforms are limited. We present the case of a 41‐year‐old woman with pleural and lymph node recurrence of type AB thymoma 3 years after robotic extended thymectomy. The patient underwent robotic‐assisted cytoreductive surgery, including complete parietal pleurectomy and resection of pleural, pericardial, and subaortic lymph node lesions, followed by HITHOC with cisplatin and doxorubicin. The procedure was performed safely, with no major perioperative complications. The patient was discharged after 7 days and remained disease‐free at 2‐year follow‐up. This case highlights the feasibility, safety, and potential oncological benefits of combining robotic cytoreductive surgery with HITHOC in the management of recurrent thymoma with pleural involvement. This minimally invasive multimodal strategy may represent a valuable option for carefully selected patients, although further studies with larger cohorts and longer follow‐up are warranted.

## 1. Case Report

Complete surgical resection is the most effective treatment of thymoma, with a survival rate of > 94% at 5 years. Thymoma recurrence occurs in 10%–30% of cases postresection, predominantly manifesting as pleural relapses (> 75%) [1, 2]. Surgical excision is a key prognostic factor for positive outcomes when combined with chemotherapy and/or radiotherapy [1]. Employing multimodal therapies prior to surgery or adjuvant therapy postresection has enhanced oncological results for patients with advanced stages [3].

Cytoreductive surgery (CRS) combined with hyperthermic intrathoracic chemotherapy (HITHOC) is an emerging technique that improves the progression‐free survival (PFS) and overall survival (OS) of patients with thymic tumors with pleural involvement [4, 5]. HITHOC offers the advantage of delivering a concentrated dose of the chemotherapy agent directly to the tumor site, minimizing systemic effects [4, 5]. Recent research has demonstrated that combining CRS with HITHOC can significantly prolong disease‐free intervals from 57 to 88 months compared with surgery alone, while also improving overall survival without substantially increasing complication rates [1–4].

Studies investigating the role of surgery and HITHOC in managing pleural dissemination of thymoma have predominantly focused on patients treated using traditional open technique [1–3]. Minimally invasive surgery could have a positive impact on the outcomes of patients eligible for removal of pleural lesions and HITHOC, thereby reducing surgical trauma, expediting recovery, and preventing delays in commencing adjuvant therapy [5]. Robotic surgery is currently the most advanced form of minimally invasive surgery, allowing complex procedures to be performed with precision. Its advanced features facilitate access to remote anatomical regions, ensuring precise exploration of the pleural space. Nonetheless, the combined application of cytoreductive robotic surgery with HITHOC has yet to be thoroughly investigated.

This case report is aimed at describing a robotic complex surgery treatment of a lymph node and pleural thymoma relapse combined with HITHOC. Approval by the Ethics Committee was obtained on August 29, 2024 (82427124.5.0000.0071) and the patient provided written informed consent.

A 41‐year‐old female patient diagnosed with a 9.4‐cm type AB thymoma underwent robotic‐assisted extended thymectomy en bloc in 2021. The pathological assessment revealed an intact capsule, absence of microscopic invasion, clear margins, and no angiolymphatic invasion.

**Figure 1:**
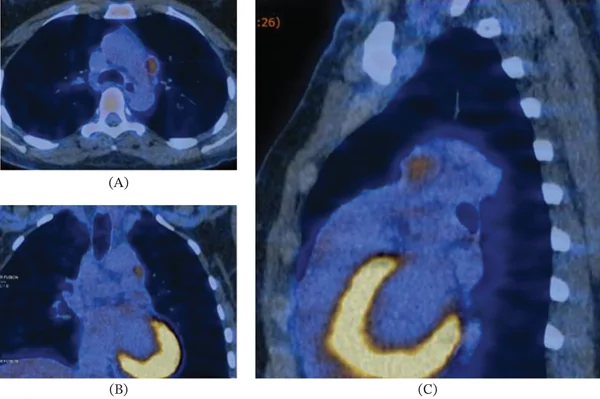
PET‐CT imaging demonstrating thymoma recurrence. (A) Axial, (B) coronal, and (C) sagittal views showing an elongated hypermetabolic nodular formation (2.2 × 1.0 cm) in the subaortic region with a maximum standardized uptake value (SUVmax) of 4.5, consistent with lymph node recurrence of thymoma.

**Figure 2:**
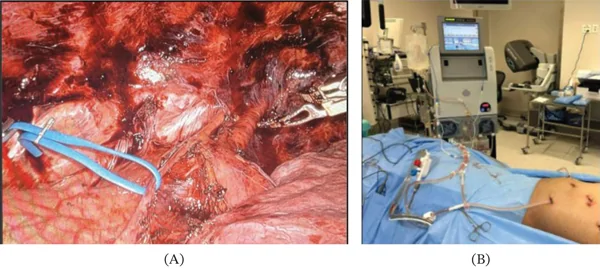
Intraoperative views of robotic cytoreductive surgery combined with hyperthermic intrathoracic chemotherapy (HITHOC). (A) Robotic endoscopic view of the pleural cavity following complete parietal pleurectomy and resection of lesions on the left visceral pleura, pericardium, and subaortic lymph node. (B) HITHOC perfusion setup using the Performer HT system (Rand, Medolla, Italy), with inflow and outflow drains connected to the perfusion circuit and temperature sensors in place, maintaining cisplatin and doxorubicin perfusion at 42°C–43°C for 60 min.

After a 3‐year follow‐up, a PET scan indicated an elongated nodular formation (2.2 × 1.0 cm) in the subaortic region with an uptake of 4.5 (Figure 1). Additionally, a smaller 0.5‐cm nodular formation in the mediastinal pleura without significant uptake and a 1‐cm lesion next to the 9th intercostal space were identified, leading to the decision for cytoreduction, surgical removal of the maximum possible tumor burden, and adjuvant HITHOC following multidisciplinary discussion. The surgical procedure was conducted utilizing a four‐access robotic daVinci Xi platform under general anesthesia and selective intubation. Complete parietal pleurectomy, *surgical removal of the membrane lining the chest wall*, was performed along with excision of lesions on the left visceral pleura, pericardium, and subaortic lymph node. Postresection, two 28‐F drains were inserted into the pleural space, with one at the apex serving as the outflow drain and the other on the diaphragm acting as the inflow drain. Temperature sensors were placed in both catheters and linked to the perfusion system before the closure of the thoracic incisions. For the HITHOC procedure, the Performer HT system (Rand, Medolla, Modena, Italy) was employed, offering automatic regulation, precise pleural space perfusion control, and the ability to achieve elevated temperatures through a specialized heat exchanger. Following the connection of the perfusion device, the pleural space was gradually filled with preloaded saline solution at 38°C, which was then raised progressively to 42.5°C before initiating the 60‐min chemotherapeutic perfusion, consisting of 50 mg/m^2^ of cisplatin and 25 mg/m^2^ of doxorubicin, based on the patient′s body surface area. Temperature was maintained with circulation flows of 400–600 mL/min with an inflow temperature range of 42°C–43°C (Figure 2). Upon completion, catheters were changed and new tubes were connected to a digital drainage system with mild suction. The patient was extubated postsurgery and spent a day in the intensive care unit. Drains were removed after 7 days and the patient was discharged. The 2‐year follow‐up scan showed no signs of recurrence.

We present an advanced surgical strategy combining robotic surgery with HITHOC for treating Stage IVa thymoma. HITHOC is increasingly recognized as a critical component of this multimodal regimen for advanced‐stage thymoma, particularly in cases with pleural or pericardial spread, even though it is not widely supported by randomized trials. The morbidity associated with the HITHOC procedure postsurgery stands at 38%, primarily related to the surgical intervention itself, yet it boasts an encouraging 5‐year survival rate of 70%–92% [4]. Furthermore, a 2023 review by the Mayo Clinic emphasized the role of HITHOC in enhancing local control of pleural pathologies, including thymoma recurrence [5]. The review also called for a standardized protocol regarding indications and procedural techniques. Advances in robotic technology have significantly expanded the feasibility of intricate procedures like pleurectomy and CRS, offering less invasive options and potentially enhancing patient recovery [3–6].

To date, the combined application of robotic CRS with HITHOC has been described in only a limited number of reports. Romano et al. published the largest available series, comprising nine patients with either pleural thymoma recurrence or early‐stage malignant pleural mesothelioma treated with robotic surgery combined with HITHOC between 2017 and 2022. Importantly, in the thymoma subgroup specifically, no intraoperative complications were recorded, the mean length of hospital stay was 6 days, and all patients remained alive after a mean follow‐up of 58 months—outcomes broadly consistent with those observed in the present case, in which the patient was discharged on postoperative day seven with no evidence of recurrence at 2 years [7]. Regarding chemotherapy protocols, Romano et al. employed cisplatin and epirubicin at 42°C for 60 min, whereas our protocol utilized cisplatin (50 mg/m^2^) and doxorubicin (25 mg/m^2^) at 42.5°C for the same perfusion duration. This variability reflects the absence of a universally standardized HITHOC regimen—a limitation acknowledged by both series and previously highlighted by the international task force convened by the Mayo Clinic [5]. The present case extends this body of evidence by describing a technically demanding scenario involving concurrent lymph node, visceral pleural, pericardial, and parietal pleural disease, all addressed within a single robotic procedure, reinforcing the potential of robotic platforms to manage complex, multicompartment thoracic recurrences with acceptable perioperative morbidity.

The decision to perform complete parietal pleurectomy rather than limited resection of the three radiologically identified lesions warrants discussion. Although imaging‐guided limited resection may appear sufficient in cases of apparently focal pleural recurrence, the biological behavior of thymoma supports a more extensive surgical approach. Pleural dissemination in thymoma is frequently characterized by microscopic implants that are undetectable on preoperative imaging, as demonstrated by studies reporting occult pleural disease in a significant proportion of patients undergoing thoracic exploration [8]. Complete cytoreduction—including pleurectomy of the parietal pleura beyond the visible nodules—is therefore justified as a strategy to eliminate potential residual microscopic disease and maximize the local oncological effect of the subsequent HITHOC perfusion, given that the chemotherapeutic agent acts on residual tumor cells exposed by the surgical resection [9, 10]. Furthermore, complete surgical resection has been consistently identified as the most important prognostic factor in pleural thymoma recurrence, and subtotal or complete pleurectomy combined with HITHOC has been associated with favorable disease‐free intervals in published series [4, 10]. In the robotic setting, the high‐definition three‐dimensional visualization and the precision of instrument articulation facilitate thorough exploration of the pleural cavity, enabling identification and resection of small implants that may escape detection during open or conventional thoracoscopic approaches [7].

A notable technical consideration in this case was the partial pericardial resection with pericardial opening required for complete excision of the pericardial nodule. Several authors have identified extensive pericardial resection requiring reconstruction as a factor warranting careful patient evaluation prior to HITHOC, given the theoretical risk of direct myocardial exposure to heated chemotherapeutic agents, particularly doxorubicin [9, 11]. In the present case, however, the pericardial defect was limited and focal, not requiring patch reconstruction, thus differing from the extensive pericardiectomy scenarios described as relative contraindications in the literature. No clinically apparent cardiac events—including arrhythmias or hemodynamic instability—were observed during the intraoperative or postoperative course, and the patient was discharged on postoperative day seven without cardiac complications. Although the absence of systematic cardiac biomarker monitoring represents a limitation of this report, the uneventful clinical course is consistent with data from series in which partial pericardial resection was performed prior to HITHOC without documented cardiac toxicity [9, 11]. These findings suggest that focal pericardial resection may not preclude safe delivery of HITHOC, though prospective studies with systematic cardiac monitoring—including troponin and electrocardiographic surveillance—are warranted to formally characterize the risk profile in this setting.

This case demonstrates that robotic CRS combined with HITHOC appears feasible and safe in carefully selected patients with pleural thymoma recurrence, offering a minimally invasive alternative to open surgery without compromising oncological efficacy. From a clinical standpoint, this strategy may translate into reduced surgical trauma, shorter hospital stay, and earlier initiation of adjuvant therapy—meaningful advantages in a population that often requires multimodal treatment. Thoracic surgeons at experienced robotic centers should consider this combined approach for patients with resectable pleural recurrence and adequate performance status. A larger prospective series with longer follow‐up is needed to establish standardized selection criteria and confirm the long‐term oncological outcomes of this technique.

## Author Contributions

All authors contributed equally.

## Funding

No funding was received for this manuscript.

## Consent

Written informed consent was obtained from the patient for publication of this case report and any accompanying images, in accordance with the ethical standards of the institutional review board (Ethics Committee Approval Number: 82427124.5.0000.0071).

## Conflicts of Interest

The authors declare no conflicts of interest.

## Data Availability

The data that support the findings of this study are available from the corresponding author upon reasonable request.
